# The first rib hypoplasia and the aberrant pulmonary artery branch detected by three-dimensional computed tomography in a surgical case with apical lung cancer, a case report

**DOI:** 10.1186/s12893-016-0199-1

**Published:** 2017-01-11

**Authors:** Yasoo Sugiura, Hiroyuki Fujimoto, Masao Naruke, Toshinori Hashizume, Shizuka Kaseda, Etsuo Nemoto

**Affiliations:** Department of General Thoracic Surgery, National Hospital Organization, Kanagawa National Hospital, 666-1 Ochiai Hadano, Kanagawa, 257-8585 Japan

**Keywords:** Rib hypoplasia, Aberrant pulmonary artery, Mediastinal basal pulmonary artery, Three-dimensional computed tomography, Case report

## Abstract

**Background:**

The complete resection is one of the most crucial requirements to achieve favorable outcomes in oncologic surgery. The apex of the lung is surrounded complicatedly by the clavicle, the first rib, the subclavian artery and vein, and the brachial plexus. Therefore, the image information especially about the infiltration of adjacent anatomic structures, facilitates the surgery in the apical lung cancer.

**Case presentation:**

A 70-year-old man presented at our hospital with a computed tomography (CT) scan showing a tumor at the left lung apex that infiltrated the chest wall. Two anatomical anomalies were found, which were the first rib hypoplasia and the aberrant pulmonary artery branch. The three-dimensional (3D) CT enhanced with using bolus tracking method, simultaneously revealed that the subclavian vessels existed between the clavicle and the second rib, and the left lingual pulmonary artery and the ventrobasal pulmonary artery diverged from the left main pulmonary artery as the first branch. We diagnosed the tumor as a primary lung squamous cell carcinoma that infiltrated the second rib, because sputum cytology suggested squamous cell carcinoma. Left lung upper lobectomy with lymph node dissection and chest wall resection (the second and third ribs) were performed with caution for the anatomical anomalies. The pathological diagnosis was pleomorphic carcinoma (5.0 × 3.0 × 1.9 cm) that invaded the second costal bone, and the pathological stage was confirmed to be pT3N0M0. Pathologically curative resection was accomplished. The patient was discharged from the hospital on 10 days after surgery.

**Conclusion:**

The 3D-CT precisely detected the anomalous structure consisted with the clavicle, the second rib, the subclavian artery and vein, the aberrant pulmonary artery branch. In the present case with the apical lung cancer, the evaluation of the anatomical structure via 3D-CT facilitated to achieve a pathological complete resection.

**Electronic supplementary material:**

The online version of this article (doi:10.1186/s12893-016-0199-1) contains supplementary material, which is available to authorized users.

## Background

Non-small-cell lung carcinoma invades the parietal pleura, soft tissue, or bone of the chest wall in 5–8% of patients undergoing surgical treatment [1]. If surgical anatomy is normal, a surgeon just has to perform operation as a textbook teaches. However, abnormal anatomy happens to make trouble to a surgeon. Due to development of various radiological survey, a surgeon preoperatively recognizes anomalies. In the present case, three-dimensional computed tomography (3D-CT) precisely determined anatomic relationship between the first rib and subclavian vein. Consequently, the achievement of a complete resection was facilitated, despite anatomical anomaly, the first rib hypoplasia and mediastinal lingular and basal pulmonary artery (PA) had been concealed.

## Case presentation

**Fig. 1:**
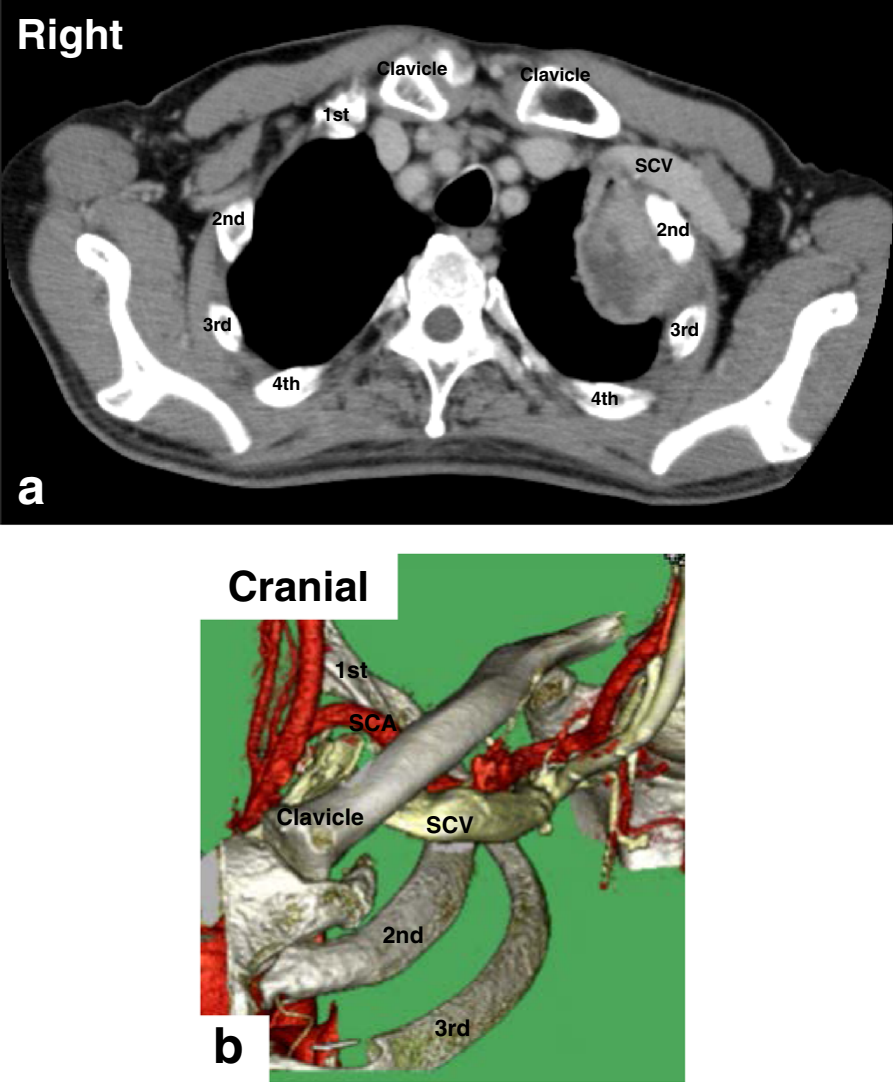
**a** Computed tomography (CT) showed the tumor infiltrating the chest wall mainly surrounding the second rib. The subclavian vein (SCV) ran between the clavicle and the second rib. **b** Three-dimensional (3D) CT clearly demonstrated the structure of thoracic apex consisted with the shorter first rib, SCV and the clavicle. The first rib did not reach the manubrium of the sternum

**Fig. 2:**
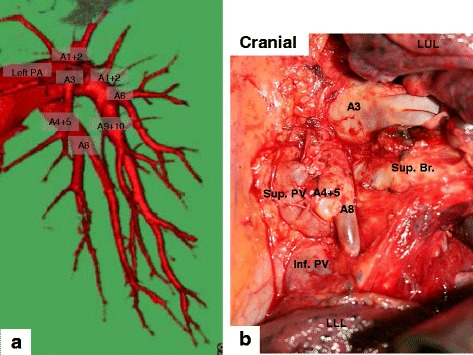
**a** 3D-CT before surgery disclosed the aberrant pulmonary artery diverged from the left main pulmonary artery as the first branch, which were the lingular artery (A4 + 5) and the ventrobasal artery (A8). **b** Intraoperative findings showed that A8 branched from the left main pulmonary artery and descended anterior to the left superior bronchus (Sup. Br.). A4 + 5 and superior pulmonary vein (Sup. PV) were cut after ligation. Inf. PV inferior pulmonary vein, LLL left lower lobe, LUL left upper lobe

A 70-year-old man was referred to our hospital because of an abnormal chest shadow around the left lung apex on a chest radiography (Additional file 1). Paralysis, paraesthesia and amyotrophy on the left arm were not recognized on the physical examination. Sputum cytology suggested squamous cell carcinoma. Enhanced CT (Toshiba Medical Systems Corporation, Aquilion ™ 64-row CT scanner) with iohexol (300 mg/mL, 100 mL, 5 mL/second) injected from the left medial cubital vein, demonstrated a 3D angiography of the PA and subclavian vein by bolus tracking method. It also showed an 8.5 cm tumor infiltrating the chest wall mainly surrounding the second rib, but did not invade the subclavian vein or brachial plexus (Fig. 1a). 3D-CT revealed that the subclavian vein and artery ran between the clavicle and the second rib because of the first rib hypoplasia (Fig. 1b). The lingular artery (A4 + 5) and the ventrobasal artery (A8) diverged from the left main PA as a first branch (Fig. 2). Fluorodeoxyglucose-positron emission tomography did not show abnormal accumulation except for the tumor at the left lung apex. We diagnosed the tumor as a primary lung squamous cell carcinoma that infiltrated the second rib. The clinical stage was IIB (T3N0M0) according to the seventh edition of the TNM classification from the Union for International Cancer Control.

Past history of the patient included a laparoscopic low anterior resection for colon cancer one month before entering our hospital, hypertension and Hashimoto disease. He had been a one-pack-a-day smoker for 40 years and had stopped smoking for 10 years.

Left lung upper lobectomy with lymph node dissection and chest wall resection were performed. The patient was placed in a lateral position for posterolateral thoracotomy. The incision at the fourth intracostal space extended toward the anterior, and the scapula was retracted cephalad with Kent retractor^Ⓡ^ (Takasago Medical Industry CO., Ltd.) to improve exposure of the apical lung cancer. The anterior and middle scalene muscles, which are usually attached to the first rib, were attached to the second rib, and the left subclavian vein was behind the anterior scalene muscle and just above the second rib. The anterior scalene muscle was divided carefully for fear of the left subclavian vein. The anterior parts of second and third ribs were divided at the costosternal junction with a wire saw. The medial parts of the second and third ribs were divided at 4 cm lateral to the extent of tumor involvement. The first rib did not reach the manubrium, and was obviously thinner. After the superior pulmonary vein was divided, A4 + 5 + 8 appeared between the superior bronchus and pulmonary vein. Subsequently, the lung resection was performed with attention to A4 + 5 + 8 diverging as the first branch from the left main PA.

The pathological diagnosis was pleomorphic carcinoma (5.0 × 3.0 × 1.9 cm) that invaded the second costal bone, and the pathological stage was confirmed to be pT3N0M0. Pathologically curative resection was accomplished. The patient was discharged from the hospital on 10 days after surgery.

## Discussion

The long-term survival rate of patients with lung cancer invading chest wall has improved, owing to the high rate of the curative resection achieved via multimodality therapy [2, 3]. Therefore, to accomplish curative resection, the precise anatomical assessment before surgery is essential.

3D-CT is not usually used to image the subclavian vein and pulmonary artery simultaneously in the thoracic surgery. On the other hand, 3D-CT is an established procedure for evaluating the anatomy of the pulmonary vessels and bronchi before lung surgery [4]. In the present case, 3D-CT showed not only the aberrant PA branch, but also the unusual position of the subclavian vein due to the first rib hypoplasia. The congenital malformations of the first thoracic rib are recognized at 0.0007–0.007% via chest radiography [5]. White et al. classified them into three types. Type I was defined that the first rib did not reach the sternal bone, which was hypoplasia. Type II was the division of ribs into vertebral and sternal bony portions. Type III was other features of anomalous first ribs [5]. The present case corresponded to type I. Several reports described that the basilar segmental PA branched from the left main PA [6, 7]. However, there is no report of case involving both the congenital first rib hypoplasia and the mediastinal lingular and basal PA. Vessels and bone are developed from mesoderm in embryology [8]. The sixth pair of pharyngeal arch arteries develops as PA from the fourth week to the eighth week of pregnancy. Rib develops from the sclerotome of somite with vertebral column originated from the paraxial mesoderm, and connects with the costal cartilage from the lateral somatic frontier during the fetal periods [8]. Thus, the coexistence of the two abnormalities seems merely a coincidence.

## Conclusion

The information of the anatomical anomaly detected using the 3D-CT facilitated to perform the lobectomy with the chest wall resection safely. Consequently, we could achieve the complete resection in the present patient of the apical lung cancer invading chest wall with the first rib hypoplasia and the aberrant PA branch.

## Additional file

Additional file 1: Original Chest X-rayR3. The chest radiography just before the patient was referred to our hospital showed the tumor at the left lungapex. (JPG 24 kb)

## Abbreviations

3D-CT: Three-dimensional computed tomography
CT: Computed tomography
PA: Pulmonary artery
