# Supplementary figures and images for: The first rib hypoplasia and the aberrant pulmonary artery branch detected by three-dimensional computed tomography in a surgical case with apical lung cancer, a case report
Source: BMC Surg. 2017 Jan 11;17:4. doi: 10.1186/s12893-016-0199-1 (PMC5225649; doi:10.1186/s12893-016-0199-1)

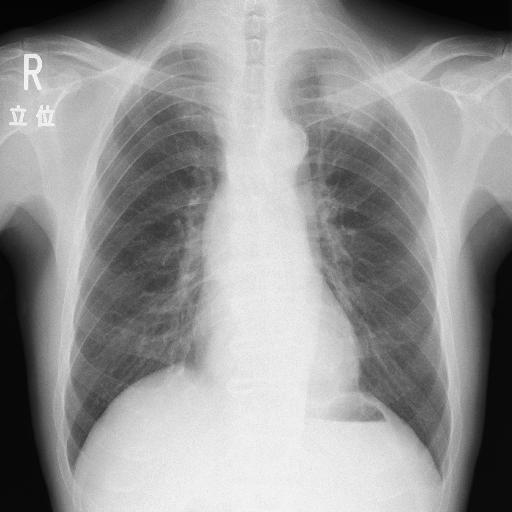

Supplement: Additional file 1: — Original Chest X-rayR3. The chest radiography just before the patient was referred to our hospital showed the tumor at the left lungapex. (JPG 24 kb) [file 12893_2016_199_MOESM1_ESM.jpg]
